# The atypical antidepressant tianeptine confers neuroprotection against oxygen–glucose deprivation

**DOI:** 10.1007/s00406-023-01685-9

**Published:** 2023-09-01

**Authors:** Burcu Ersoy, Marie-Louise Herzog, Wen Pan, Simone Schilling, Matthias Endres, Ria Göttert, Golo D. Kronenberg, Karen Gertz

**Affiliations:** 1 Department of Neurology, Charité-Universitätsmedizin Berlin, Corporate Member of Freie Universität Berlin and Humboldt-Universität zu Berlin, Berlin, Germany; 2 Center for Stroke Research Berlin, Department of Experimental Neurology, Charité-Universitätsmedizin Berlin, Corporate member of Freie Universität Berlin and Humboldt-Universität zu Berlin, Berlin, Germany; 3 Berlin-Brandenburg School for Regenerative Therapies, Charité-Universitätsmedizin Berlin, Berlin, Germany; 4 DZHK (German Center for Cardiovascular Research), Partner site, Berlin, Germany; 5 Berlin Institute of Health at Charité, Universitätsmedizin Berlin, Berlin, Germany; 6 Einstein Center for Neurosciences, Charité-Universitätsmedizin Berlin, Berlin, Germany; 7 DZNE (German Center for Neurodegenerative Diseases), Partner site, Berlin, Germany; 8 DZPG (German Center for Mental Health), Partner site, Berlin, Germany; 9 Department of Psychiatry, Psychotherapy and Psychosomatics, University Hospital of Psychiatry Zürich, Lenggstrasse 31, P.O. Box 363, 8032 Zurich, Switzerland

**Keywords:** Tianeptine, Ischemia, Stroke, Neuroprotection, Serotonin

## Abstract

Proregenerative and neuroprotective effects of antidepressants are an important topic of inquiry in neuropsychiatric research. Oxygen–glucose deprivation (OGD) mimics key aspects of ischemic injury in vitro. Here, we studied the effects of 24-h pretreatment with serotonin (5-HT), citalopram (CIT), fluoxetine (FLU), and tianeptine (TIA) on primary mouse cortical neurons subjected to transient OGD. 5-HT (50 μM) significantly enhanced neuron viability as measured by MTT assay and reduced cell death and LDH release. CIT (10 μM) and FLU (1 μM) did not increase the effects of 5-HT and neither antidepressant conferred neuroprotection in the absence of supplemental 5-HT in serum-free cell culture medium. By contrast, pre-treatment with TIA (10 μM) resulted in robust neuroprotection, even in the absence of 5-HT. Furthermore, TIA inhibited mRNA transcription of candidate genes related to cell death and hypoxia and attenuated lipid peroxidation, a hallmark of neuronal injury. Finally, deep RNA sequencing of primary neurons subjected to OGD demonstrated that OGD induces many pathways relating to cell survival, the inflammation-immune response, synaptic dysregulation and apoptosis, and that TIA pretreatment counteracted these effects of OGD. In conclusion, this study highlights the comparative strength of the 5-HT independent neuroprotective effects of TIA and identifies the molecular pathways involved.

**Supplementary Information:**

The online version contains supplementary material available at 10.1007/s00406-023-01685-9.

## Introduction

Beyond treating affective disorders, antidepressants represent promising neuroprotective drugs for the treatment of ischemic stroke. Animal stroke studies that have employed the middle cerebral artery occlusion model (MCAo) and several clinical trials in stroke patients have demonstrated the neuroprotective effects of antidepressants, particularly those belonging to the selective serotonin reuptake inhibitors (SSRIs) [41, 51, 66]. The neuroprotective effects of antidepressants in animal models of stroke are evidenced by a reduction in infarct volume, improvement in motor function, promotion in neurogenesis and restoration of plasticity [20, 40, 44, 49]. This effect has been demonstrated in clinical trials with stroke patients by improving recovery of motor functions and neurological deficits independent of depression [9, 53].

The SSRIs fluoxetine (FLU) and citalopram (CIT) have been extensively studied, including the studies mentioned above. In contrast, tianeptine (TIA) is a selective serotonin reuptake enhancer (SSRE) that increases serotonin (5-HT) reuptake in the brain. Thus, TIA contradicts the traditional monoaminergic hypothesis of depression, and its particular neurobiological properties set it apart from SSRIs [52, 72]. Numerous research studies have shown that TIA normalizes stress-induced neuronal changes and glutamatergic neurotransmission and reduces apoptosis, particularly in the hippocampus [12, 38, 48]. Moreover, TIA supports the ability to improve synaptic plasticity and neurogenesis impaired by stress and acts as a potent antidepressant, supporting the neuroplasticity hypothesis of depression [23, 72]. However, the precise neuroprotective mechanisms by which different classes of antidepressants, including TIA, mediate neuroprotection against brain injury are not fully understood.

In this study, we employed the oxygen–glucose deprivation (OGD) model, an established in vitro model of stroke. OGD can be used to reproduce complex processes of neuroinflammation, neurodegeneration, and neuroexcitotoxicity, which also play an important role in ischemic damage. This also makes OGD particularly suitable for studying neuroprotective mechanisms [6, 10, 29, 68]. We examined the effects of 5-HT, CIT, FLU, and TIA on primary neuronal cultures (PNCs) subjected to transient OGD/reoxygenation with the aim of dissecting cell autonomic responses to ischemic injury. Surprisingly, TIA showed a more pronounced neuroprotective effect than the other compounds. This prompted us to conduct a more comprehensive analysis of the molecular mechanisms responsible for this neuroprotective effect by using deep RNA sequencing. The results showed that TIA suppresses the deleterious effects of OGD, particularly by regulating genes related to calcium and p53 signaling pathways. Overall, we postulate a 5-HT-independent neuroprotective mechanism of TIA action that rescues cell viability and reduces lipid peroxidation in cortical neurons under ischemic conditions.

## Materials and methods

### Treatments and OGD

Primary neuronal cultures (PNCs) were obtained from the cortex of fetal mice at embryonic day 15 as described [42]. Neurons at day in vitro (DIV) 8 were used to study concentration-dependent toxicity of CIT, FLU, and TIA treatments. Antidepressants were administered at defined concentrations (0.1, 1, 10, 100 μM). Cells were incubated in a concentration series of each antidepressant for 24 h to determine the optimal concentration for each compound (Supplementary Fig. 1). Accordingly, PNCs at DIV 8 were used for the treatments as follows: vehicle (0.1% DMSO), 5-HT (50 μM), CIT (10 μM), FLU (1 μM), TIA (10 μM), CIT + 5-HT, FLU + 5-HT, and TIA + 5-HT. 24 h after the initiation of drug treatment, i.e., at DIV 9, 24- and 6-well plates with 0.3 × 10^6^ and 1.5 × 10^6^ cells per well, respectively, were exposed to 2–3 h of OGD. OGD was performed as described previously [19]. The plates were incubated in a CO2 incubator for 24-h for cell viability/metabolic assays and with 6-h reoxygenation for mRNA analysis.

### Cell viability and cell toxicity assays

**Fig. 1:**
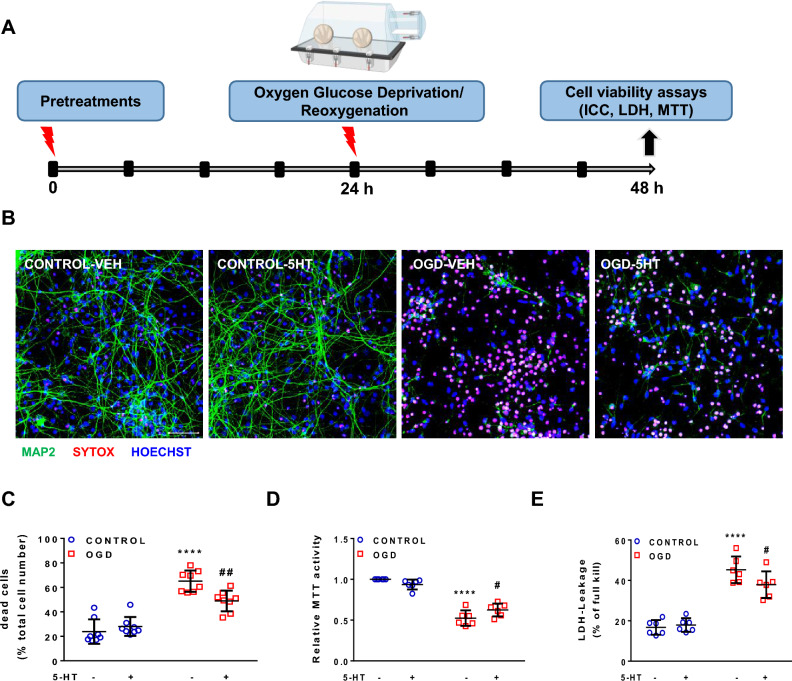
Neuroprotective effect of 5-HT in primary neuronal cultures exposed to 2-h OGD **A** Schematic diagram of the experimental design. 5-HT was applied at a concentration of 50 μM. **B** Representative immunofluorescence images of primary neuronal cultures stained with neuronal marker MAP2 (green), cell death marker SYTOX (red), and nuclear marker Hoechst 33,258 (blue) 24 h after OGD. Scale bar, 50 µm (all panels). **C** Quantitative analysis of immunofluorescence stainings. N = 8 biological replicates per group. Two-way ANOVA with Holm-Sidak’s multiple-comparison test; ****p < 0.0001 OGD-VEH versus CONTROL-VEH, ^##^*p* < 0.01 OGD-5-HT versus OGD-VEH. **D**, **E** Cell death was studied at 24 h after 2-h OGD using MTT and LDH assays. **D** MTT values were normalized to the CONTROL-VEH condition. **E** The LDH values reflect the ratio of the experimental absorbance value to the full-kill value. N = 6 biological replicates per group. Two-way ANOVA with Holm-Sidak’s multiple-comparison test; ****p < 0.0001 OGD-VEH versus CONTROL-VEH; ^#^*p* < 0.05 OGD-5-HT versus OGD-VEH

**Fig. 2:**
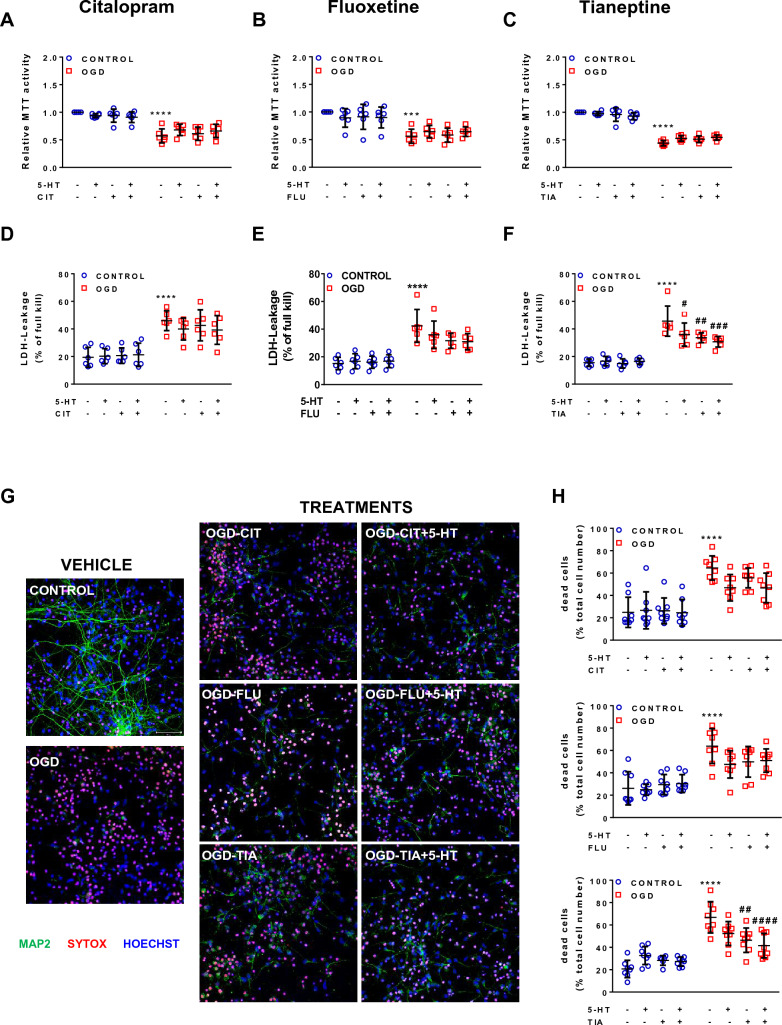
Neuroprotective effect of CIT, FLU and, TIA in primary neuronal cultures exposed to 2-h OGD **A**–**F** Experimental drugs were applied 24 h before OGD. Drugs were applied in the following concentrations: CIT, 10 μM; FLU, 1 μM; TIA, 10 μM. 5-HT was applied at a concentration of 50 μM. **A**–**C** The MTT assay was carried out to examine neuronal viability at 24 h after 2-h OGD. MTT values were normalized to the CONTROL-VEH condition. **D**–**F** Similarly, the LDH assay was performed to study the effects of CIT, FLU, and TIA on the structural integrity of neurons at 24 h after 2-h OGD. LDH values reflect the ratio of the experimental absorbance value to the full-kill value. N = 6 biological replicates per group. Two-way ANOVA with Holm-Sidak’s multiple-comparison test; ****p < 0.0001 OGD-VEH versus CONTROL-VEH, ^#^*p* < 0.05, ^##^*p* < 0.01, ^###^*p* < 0.001 OGD-treatment versus OGD-VEH. **G** Primary neuronal cultures were stained with neuronal marker MAP2 (green), cell death marker SYTOX (red), and nuclear marker Hoechst 33,258 (blue). Scale bar, 50 µm (all panels). **H** Quantitative analysis of immunofluorescence stainings. N = 6–8 biological replicates per group. Two-way ANOVA with Holm-Sidak’s multiple-comparison test; ****p < 0.0001 OGD-VEH versus CONTROL-VEH, ^##^*p* < 0.01, ^####^*p* < 0.0001 OGD-treatment versus OGD-VEH

Cell viability was measured by evaluating mitochondrial activity of the cells through MTT (3-(4,5-dimethylthiazol-2-yl)-2,5-diphenyltetrazolium bromide) assay. MTT was added into each well at a ratio of 1:10. Cells were incubated for 40 min at room temperature (RT). Subsequently, 10% SDS was added into each well and the samples were incubated overnight. The absorbance of formazan produced by the cells was measured at 550 nm with a microplate reader (2–3 wells per condition in Fig. 1D, 1 well per condition in Fig. 2A–C).

In addition, cytotoxicity was evaluated by lactate dehydrogenase (LDH) release into the medium using a previously published protocol [22]. After normalization of the values with LDH standards, the ratio of absorbance to the absorbance after full-kill at 340 nm gives the proportion of dead cells (2–3 wells per condition in Fig. 1E, 1 well per condition in Fig. 2D−F).

### Immunocytochemistry (ICC)

Prior to fixation of PNCs, SYTOX staining was performed to label dead cells. SYTOX stain (excitation/emission: 546/570 nm; ThermoFisher) diluted 1:5000 in live cell imaging solution was added to the cells and incubated for 15 min at RT. Cells were then fixed in 4% paraformaldehyde overnight as described [27]. The following antibodies were used: mouse monoclonal anti-MAP2 (1:200; Sigma) and donkey anti-mouse Alexa 488 (1:400; Invitrogen). Hoechst 33,258 stain (1:1000) was used to label nuclei.

Images were taken using confocal laser scanning microscopy (LSM 700, Zeiss) under 20X magnification. The number of SYTOX + and Hoechst + cells was assessed using ImageJ software (NIH, Bethesda, USA). The mean cell count from 2–4 different images per well per condition was calculated (3 wells per condition in Fig. 1C, 1 well per condition in Fig. 2H). The ratio of SYTOX + cell numbers to the Hoechst + cell numbers in corresponding images was used to quantify the percentage of dead cells.

### RNA isolation and quantitative real-time polymerase chain reactions (qRT-PCR)

Total RNA from each sample was extracted using the NucleoSpin® XS Kit following the manufacturer’s guidelines (MACHEREY–NAGEL). The RNA was reverse transcribed to cDNA using 300 U M-MLV Reverse Transcriptase (Promega Corp.), 20 U RNasin® ribonuclease inhibitor (Promega Corp.), 1.96 mM DTT (Promega Corp.), 0.147 mM of each dNTP (Promega Corp.), 5.88 µM random primers (Roche Diagnostics), and incubated in a thermocycler for 5 min at 21 °C, for 1 h at 37 °C and 5 min at 95 °C. The cDNA underwent PCR amplification using the LightCycler® 480 and lightCycler 480 SYBR Green I Master-Kit (Roche Diagnostics). Gene specific primer sequences are given in Table 1. PCR conditions were used as described previously [77]. Quantification of cDNA is relative to housekeeping gene, *Reep5* [54].

**Table 1:** Specific primer sequences used in qRT-PCR

Gene	Forward primer	Reverse primer
*Trp53*	GAC AGC CAA GTC TGT TAT GTG C	GTC TTC CAG ATA CTC GGG ATA C
*Serpine1*	GCA TAC CAA AGG TAT GAT CAG TG	GGT GAA CTC AGT GTA GTT GAA C
*Mmp3*	GAA CGA TGG ACA GAG GAT GTC AC	GAC TGG GTA CAT CAG AGC TTC AG
*Adamts7*	GCA AGT GGA GAG CTA TGT GCT G	CAA ATC CTT CCT GGT GAG CAG G
*Hif1a*	CAC ACA GAA ATG GCC CAG TGA G	CAG TGA AGC ACC TTC CAC GTT G
*Htr1a*	CAG GTG CTC AAC AAG TGG ACT C	CAA TGA GCC AAG TGA GCG AGA TC
*Htr1b*	GCT CTC CAA CGC CTT TGT AAT C	CAT GAT GGA AGC AGT GCA ACA G
*Htr2c*	CCT AGC CAT TGC TGA TAT GCT G	CGA ATT GAA CCG GCT ATG CTC A
*Htr4*	GCT AAT GTG AGT TCC AAC GAG G	GAT GTC TTG GAC CAG CTC AAT G
*Htr6*	GTG CCA TCT GCT TCA CCT ACT G	CTA CTG TCA GCA GAC TCC ATC C
*Htr7*	CTG TGC GTG ATC AGC ATC GAC A	CAC ATT CTG AGC CCA TCC GAA G
*Reep5*	CTG ATA GGT TTC GGA TAC CCA G	GAC TCG TGC TTG AGG AAG ATA G

### Assessment of lipid peroxidation

A ratiometric fluorescent based lipid peroxidation assay was performed following the manufacturer’s guidelines (ab243377 Lipid Peroxidation Assay Kit, Abcam). Fluorescence intensities were measured using ImageJ software (NIH, Bethesda, USA).

### RNA-seq expression analysis

Total RNA was extracted 6 h after 3-h OGD from 4 biological replicates of each group (PNCs of CONTROL-VEH, CONTROL-TIA, OGD-VEH and OGD-TIA treatment groups). Assessment of RNA quality, cDNA synthesis, library preparation, sequencing, data pre-processing, transcript alignment to a reference genome (Mus_musculus.GRCm38.92.gtf), estimation of gene and isoform expression, principal component analysis (PCA), heatmap of the sample-to-sample distances and differential gene expression analysis were conducted by ATLAS Biolabs GmbH (Berlin, Germany) with the tools listed in Table 2. Libraries were prepared from total RNA using Illumina TruSeq Stranded mRNA Library Prep Kit and sequenced with 50 single-end reads. 18–23 million reads per sample were aligned to the reference genome.

**Table 2:** The tools used in RNA-seq analysis

Analysis	Tools	Short description	Detail description	More
Fastq quality control	fastqc	quality control checks on raw sequence data coming from high throughput sequencing pipelines	FastQC	
Mapping	STAR	STAR (Spliced Transcripts Alignment to a Reference) is an RNA-seq mapper that performs highly accurate spliced sequence alignment at an ultrafast speed	STAR: ultrafast universal RNA-seq aligner	
Estimation of gene and isoform expression	eXpress	a tool for quantification of RNA-Seq data	Streaming fragment assignment for real-time analysis of sequencing experiments	Comparative evaluation of isoform-level gene expression estimation algorithms for RNA-seq and exon-array platforms
Differential gene expression	DESeq2	Estimate variance-mean dependence in count data from high-throughput sequencing assays and test for differential expression based on a model using the negative binomial distribution	Moderated estimation of fold change and dispersion for RNA-seq data with DESeq2	

Differential expression analysis was performed using DESeq2, which is a R/Bioconductor package adopting Benjamin-Hochberg correction [47]. Genes with an adjusted p-value (padj; false discovery rate corrected p-value) ≤ 0.05 and log2 fold change ≥ 1 or ≤ − 1 (the fold-change is the ratio of the mean normalized intensities for the two conditions) were considered to be differentially expressed genes (DEGs). Venn diagrams were generated with a web-based tool called BioVenn (https://www.biovenn.nl/) [31]. The expression levels of the DEGs (from OGD-VEH vs. CONTROL-VEH) were visualized with heatmapper (http://www.heatmapper.ca) according to their log2(TMM FPKM) values in all groups. RNA-seq data were deposited at the Gene Expression Omnibus (GEO) under GEO accession number GSE234798.

### Pathway analysis

Pathway enrichment analysis was performed using g:Profiler (version e94_eg41_p11_6f51822) to identify significant pathways involving differential gene expression according to Kyoto Encyclopedia of Genes and Genomics (KEGG) with Bonferroni method to adjust for multiple testing [59]. Pathways with an adjusted p-value ≤ 0.05 were considered to be significantly enriched.

### Gene ontology (GO) analysis

GO enrichment analysis was performed using g:Profiler (version e94_eg41_p11_6f51822) to identify significant GO terms involving differential gene expression with Bonferroni correction to adjust for multiple testing [59]. GO terms with an adjusted p-value ≤ 0.05 were determined to be significantly enriched. As a last step, REVIGO was used to summarize the list of GO terms by filtering out redundant GO terms [69].

### Statistics

Experiments were carried out in a blinded fashion. Data are presented as individual data points with mean ± SD. Unless otherwise indicated, groups were compared by ANOVA with level of significance set at 0.05 and two-tailed *p*-values. Statistical analysis was performed using GraphPad Prism version 6.00 (GraphPad Software, Inc.).

## Results

### Neuroprotective effect of 5-HT in primary neuronal cultures exposed to 2-h OGD

A schematic diagram of the experimental design is given in Fig. 1A. Briefly, to generate an in vitro model of brain ischemia, PNCs were subjected to OGD for 2 h, followed by 24-h reoxygenation. To ascertain the effects of 5-HT and three different antidepressants—two of which are SSRIs (CIT and FLU); and one has been described as SSRE (TIA) [73]—the pre-treatments were initiated 24 h before OGD. The effects on cell viability were analyzed 24 h after OGD using ICC as well as MTT and LDH assays.

ICC was performed with an antibody directed against the neuronal marker microtubule associated protein-2 (MAP2) [45], cell death marker SYTOX Orange, and nuclear stain Hoechst 33,258. Histological analysis confirmed that 2-h OGD massively increased cell death with most dendrites disappearing in the following 24 h (Fig. 1B). Quantitative analysis further demonstrated that 5-HT (50 μM) pretreatment decreased cell death by around 25% (Fig. 1C). In addition, 5-HT pretreatment significantly increased MTT activity as a marker of cell viability by approximately 18% (Fig. 1D) and decreased LDH leakage by approximately 16% (Fig. 1E) 24 h after OGD, indicating less cell death. To explore how PNCs might be responsive to 5-HT treatment, we studied mRNA expression of 5-HT receptor subtypes *Htr1a*, *Htr1b*, *Htr2c*, *Htr4*, *Htr6,* and *Htr7.* RT-PCR confirmed the presence of all receptors studied (data not shown). A significant decrease in the mRNA level of *Htr1a* was observed while there was no change in the level of *Htr1b* at 6 h of reoxygenation following 3-h OGD (Supplementary Fig. 2).

### Neuroprotective effect of CIT, FLU, and TIA in PNCs exposed to 2-h OGD

First, concentration-dependent toxicity of CIT, FLU, and TIA was evaluated through MTT assay after 24 h of treatment (range from 0.1 to 100 μM; Supplementary Fig. 1). We observed a statistically significant decrease in cell viability at the concentration of 100 μM for all three compounds. In addition, we observed a slight decrease in cell viability at the concentration of 10 μM FLU. Consequently, the final concentrations chosen for the antidepressants in the following experiments were 10 μM CIT, 10 μM TIA, and 1 μM FLU.

PNCs exposed to 2-h OGD and sham PNCs were pretreated with CIT, FLU, and TIA either alone or in combination with 5-HT (Fig. 2A–F). OGD caused a statistically significant decrease in MTT activity (Fig. 2A–C) together with increased LDH leakage (Fig. 2D–F). At the concentrations used, CIT and FLU (either in the absence or presence of 5-HT) did not show a statistically significant effect in MTT and LDH assays. TIA, on the other hand, irrespective of whether used alone or in combination with 5-HT, significantly reduced LDH leakage (by about 26% of OGD-VEH) (Fig. 2F). The combined effect of TIA and 5-HT was also quite remarkable with a further decrease of LDH leakage by about 32% of OGD-VEH (Fig. 2F).

Triple labeling of PNCs subjected to OGD or sham treatment and treated with vehicle, CIT, FLU, and TIA with or without 5-HT confirmed that OGD decreased the number of MAP2-positive cells and increased the number of SYTOX + cells (Fig. 2G). While the percentage of dead cells in the CIT and FLU conditions appeared somewhat reduced relative to the OGD-VEH condition, these changes did not reach statistical significance. By contrast, the neuroprotective effect of TIA was readily apparent (Fig. 2H). TIA, even in the absence of 5-HT, resulted in the strongest decrease in cell death and axon atrophy, suggesting it is the most effective antidepressant among these three in promoting the survival of PNCs exposed to OGD.

### Effect of OGD and TIA treatment on the modulation of gene expression and lipid peroxidation

**Fig. 3:**
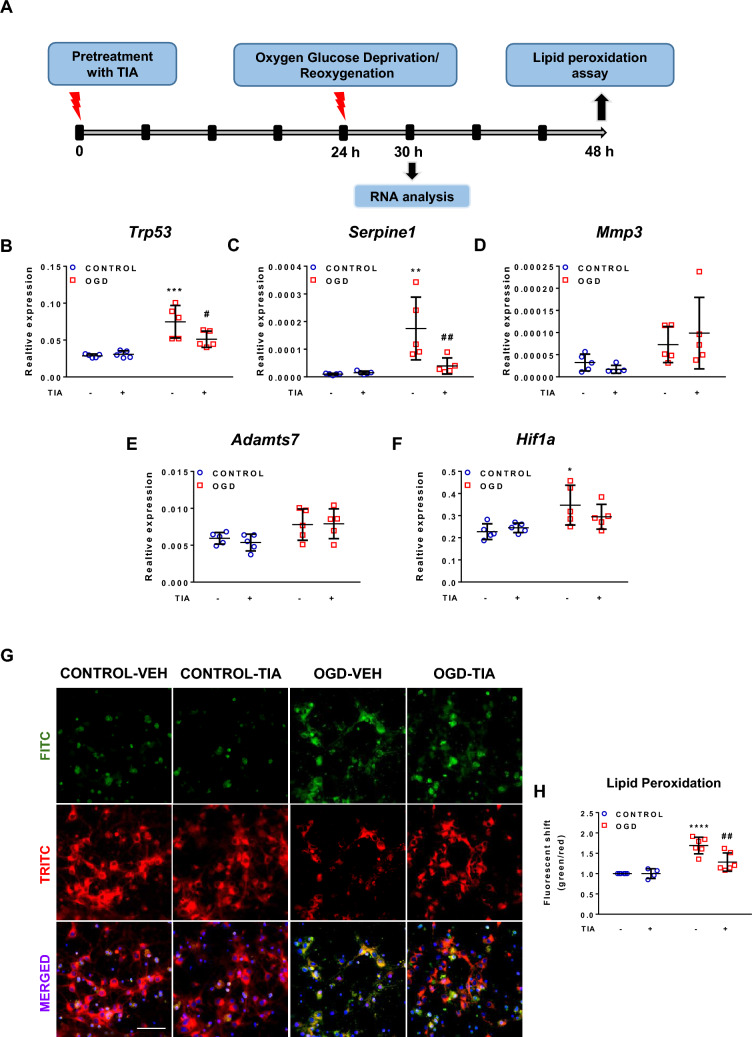
Effects of OGD and TIA on gene expression and lipid peroxidation in primary neuronal cultures **A** Schematic diagram of the experimental design. TIA was applied at a concentration of 10 μM **B**–**F** mRNA transcription of candidate genes transformation-related protein 53 (*Trp53*), plasminogen activator inhibitor-1 (*Serpine1*), matrix metalloproteinase-3 (*Mmp3*)**,** a disintegrin and metalloproteinase with thrombospondin motifs 7 (*Adamts7*), and hypoxia inducible factor 1a (*Hif1a*). Values were normalized to housekeeping gene Reep5. N = 5 biological replicates per group. Two-way ANOVA with Holm-Sidak’s multiple-comparison test; *p < 0.05, **p < 0.01, ***p < 0.001 OGD-VEH versus CONTROL-VEH; ^#^*p* < 0.05, ^##^*p* < 0.01 OGD-TIA versus OGD-VEH. **G** Fluorescence images of primary neuronal cultures stained with ratiometric lipid peroxidation sensor R590/G525 and nuclear marker Hoechst (blue). Scale bar, 50 µm (all panels). **H** Lipid peroxidation was quantified by measuring the ratio of fluorescence intensities at Ex/Em 490 nm/530 nm (FITC) and 545 nm/600 nm (TRITC). Each data point represents the ratio of green fluorescence to red fluorescence normalized to the CONTROL-VEH condition. N = 4–6 biological replicates per group. Two-way ANOVA with Holm-Sidak’s multiple-comparison test. ****p < 0.0001 OGD-VEH versus CONTROL-VEH, ^##^*p* < 0.01 OGD-TIA versus OGD-VEH

Having found that TIA exerts more potent neuroprotective effects than either CIT or FLU against OGD, we further characterized the neuroprotection conferred by TIA by studying mRNA transcription and the production of reactive oxygen species (ROS). The schematic of these experiments is given in Fig. 3A. To evaluate the effects of OGD and TIA treatment on mRNA expression in a candidate approach, neuronal cells were pretreated with TIA alone for 24 h before 3-h OGD. After 6-h reoxygenation, mRNA transcription of candidate genes transformation-related protein 53*** (****Trp53)*, plasminogen activator inhibitor-1* (Serpine1)*, matrix metalloproteinase-3 *(Mmp3)***,** a disintegrin and metalloproteinase with thrombospondin motifs 7 *(Adamts7)*, and hypoxia inducible factor 1a *(Hif1a)* was analyzed (Fig. 3B–F). In accordance with the cell viability results reported above, OGD caused a more than twofold increase in the expression of cell death marker *Trp53.* TIA treatment significantly counteracted this effect of OGD (Fig. 3B). Serpine1, which is an established mRNA marker for hypoxia [39], was also strongly upregulated after OGD (by about tenfold). Again, this upregulation was significantly decreased by TIA (Fig. 3C). *Mmp3* and *Adamts7* mRNA expressions were not significantly impacted by either OGD or TIA (Fig. 3D, E). Finally, although *Hif1a* mRNA was increased after OGD, it was not influenced by TIA treatment (Fig. 3F). As a global criterion of oxidative stress, lipid peroxidation in PNCs was quantified 24 h after 2-h OGD. Fluorescent images of live cells showed that OGD caused increased lipid peroxidation. This increase in lipid peroxidation was significantly attenuated by TIA treatment relative to the OGD-VEH group, which is again consistent with cell viability results (Fig. 3G, H).

### Effects of OGD and TIA treatment on the transcriptomic profiles of PNCs

Next, RNA-Seq was used to study the transcriptomic effects of OGD and TIA treatment. Neuronal cells were pretreated with TIA for 24 h before 3-h OGD. At 6-h of reoxygenation, the transcriptomic profiles of 4 replicates of each group (CONTROL-VEH, CONTROL-TIA, OGD-VEH and OGD-TIA) were analyzed. Gene expression levels were determined for each condition. Sample to sample distances for the four comparisons are given as a heatmap in supplementary Fig. 3A.

**Fig. 4:**
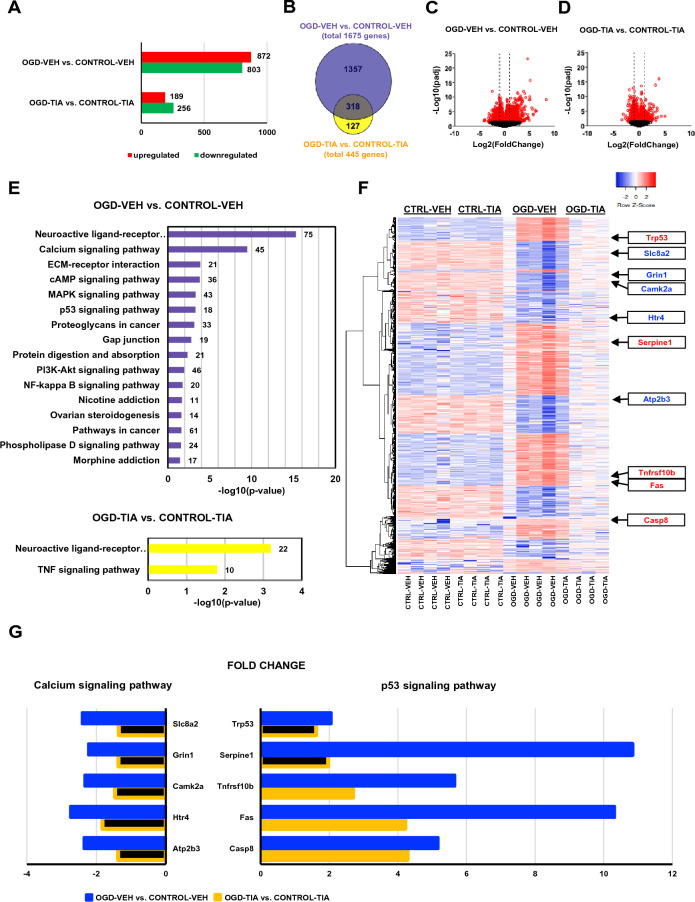
Transcriptomic analysis **A**–**G** Primary neuronal cultures were pretreated with 10 μM TIA or vehicle 24 h before 3-h OGD or sham. Total RNA was isolated at 6 h of reoxygenation for transcriptomic analysis (n = 4 biological replicates per group). **A** The bar graph illustrates the number of upregulated and downregulated genes for OGD-VEH versus CONTROL-VEH and OGD-TIA versus CONTROL-TIA (padj ≤ 0.05 and fold change ≥ 2). **B** Venn diagram showing the number of differentially expressed genes (DEGs) for the comparison of OGD versus CONTROL in the absence (purple circle) and presence (yellow circle) of TIA. **C**, **D** Volcano plots of all genes. Log2 of the fold changes between OGD-VEH and CONTROL-VEH **(C)** and between OGD-TIA and CONTROL-TIA **D** are plotted in the x axis while the negative log10 of the padj values are plotted in the y-axis. Red dots in **(C)** and **(D)** represent the genes which have padj values ≤ 0.05 for the comparison between OGD and CONTROL. The red circles outside of the dashed lines represent DEGs. Dashed lines are set to log2 (foldchange) =  ± 1. **E** Pathway enrichment analysis. The number of DEGs is given for each pathway. Significantly enriched pathways in OGD-VEH versus CONTROL-VEH (top) and in OGD-TIA versus CONTROL-TIA (bottom). **F** Clustered heatmap of the all DEGs (according to OGD-VEH versus CONTROL-VEH) were generated based on their degree of expression similarity in all groups (Euclidean distance, complete linkage). **G** Expression changes of *Slc8a2*, *Grin1*, *Camk2a*, *Htr4, Atp2b3* from calcium signaling pathway and *Trp53*, *Serpine1*, *Tnfrsf10b*, *Fas, Casp8* from p53 signaling pathway in the comparisons of OGD-VEH versus CONTROL-VEH (blue) and OGD-TIA versus CONTROL-TIA (yellow). The expression changes of black-filled genes are not statistically significant according to the criteria padj ≤ 0.05 and fold change ≥ 2 for the corresponding comparison

We identified 1675 genes that were differentially expressed (with padj ≤ 0.05 and log2 fold change ≥ 1 or ≤ − 1) between OGD-VEH and CONTROL-VEH (872 upregulated and 803 downregulated), and 445 genes that were differentially expressed between OGD-TIA and CONTROL-TIA (189 upregulated and 256 downregulated) (Fig. 4A). The number of genes which were differentially regulated by OGD irrespective of TIA treatment was 318 (intersection area of the two circles in the Venn diagram; Fig. 4B). TIA decreased the number of DEGs between the OGD and the CONTROL condition (Fig. 4B). The expression changes of all genes were visualized by volcano plots (Fig. 4C, D).

Next, we performed a pathway enrichment analysis to identify significant pathways involving DEGs for the comparison of OGD-VEH and CONTROL-VEH and for the comparison of OGD-TIA and CONTROL-TIA (Fig. 4E). 16 KEGG enrichment pathways [34, 35] were obtained for the first comparison. The neuroactive ligand-receptor interaction pathway, which is associated with cell survival, was regulated by OGD both in the presence and in the absence of TIA. Crucial pathways involved in the inflammatory response and apoptosis (such as MAPK signaling pathway, p53 signaling pathway, PI3K-Akt signaling pathway), and cellular communication (such as calcium signaling, the extracellular matrix-receptor interaction, cAMP signaling pathway, gap junction pathway) were all regulated in OGD-VEH vs. CONTROL-VEH (Fig. 4E, top). However, interestingly, these pathways disappeared with TIA pretreatment (Fig. 4E, bottom). Conversely, OGD activated the TNF signaling pathway only in the presence of TIA. In addition, we performed GO enrichment analysis [58]. The top significant GO terms are given in Supplementary Fig. 3B–C.

To further investigate the treatment-dependent gene expression patterns, we applied hierarchical clustering of the DEGs (of the OGD-VEH vs. CONTROL-VEH) across all experimental groups (Fig. 4F). An overall pattern of differences in gene expression is readily apparent with OGD-VEH contrasting with both CONTROL-VEH and OGD-TIA. Generally speaking, DEGs either show upregulation caused by OGD and inhibition of this upregulation by TIA (i.e., genes in red), or downregulation caused by OGD and a reduction of this downregulation as an effect of TIA (i.e., genes in blue). Genes associated with the generation of reactive oxygen species, the inflammatory response and cell death such as TNF receptor superfamily member 6 *(Fas)*, Trp53*,* Serpine1*,* tumor necrosis factor receptor superfamily member 10b *(Tnfrsf10b*), caspase 8 *(Casp8*) [8, 37, 74] showed upregulation in the OGD-VEH condition and inhibition of this upregulation in the OGD-TIA condition. Interestingly, these genes are all members of the p53 signaling pathway. Genes associated with neurotransmission, synaptic plasticity and neuronal survival such as calcium/calmodulin-dependent protein kinase II alpha *(Camk2a*), solute carrier family 8 *(Slc8a2*), which encodes for the Na^+^*/*Ca^2+^ exchanger (NCX), glutamate receptor ionotropic NMDA1 (*Grin1)*, 5-hydroxytrptamine (serotonin) receptor 4 *(Htr4*) and *Atp2b3* (*ATPase*), which encodes for plasma membrane Ca^2+^ ATPase (PMCA) [4, 26, 28, 33, 46] showed the opposite pattern. These latter genes are all related to calcium signaling, the top pathway whose regulation was lost when TIA was applied. Supplementary Fig. 4 summarizes our transcriptomic results regarding p53- and calcium signaling.

The expression changes of *Trp53*, *Slc8a2*, *Grin1*, *Camk2a*, *Htr4*, *Serpine1*, *Atp2b3*, *Tnfrsf10b, Fas and Casp8* for the comparisons of OGD-VEH vs. CONTROL-VEH and OGD-TIA vs. CONTROL-TIA are given in Fig. 4G. In the absence, but not in the presence, of TIA, the DEGs related to calcium signaling were all downregulated in OGD vs. CONTROL. DEGs in the p53 signaling pathway were similarly all upregulated in OGD-VEH vs. CONTROL-VEH. Although *Tnfrsf10b, Fas* and *Casp8* were still significantly upregulated in response to OGD even when TIA was applied, the magnitude of upregulation was much lower. Of noteworthy importance, *Trp53 and Serpine1* were no longer significantly altered by OGD upon TIA pretreatment.

Taken together, we demonstrate that OGD activates many pathways related to cell survival, inflammation, lipid peroxidation, synaptic dysregulation and apoptosis, and that, broadly speaking, TIA pretreatment, by preconditioning of the primary neurons, inhibits these effects of OGD.

## Discussion

This study yielded the following major findings: (1) 24-h 5-HT (50 μM) pre-treatment of primary mouse cortical neurons significantly decreased cell death following 2-h OGD. This report, therefore, demonstrates the neuroprotective action of 5-HT in OGD, i.e., a cell-culture model of ischemic injury. (2) Interestingly, the antidepressant tianeptine (TIA; 10 μM) also showed statistically significant neuroprotective effects against OGD that could even be observed in the absence of 5-HT. By contrast, 24-h pretreatment with two SSRIs (CIT and FLU) did not confer neuroprotection. (3) TIA pretreatment decreased lipid peroxidation and mRNA levels of cell death and hypoxia markers (*Trp53*, *Serpine1*) in OGD exposed primary cortical cells. (4) Transcriptomic profiling of neurons subjected to OGD demonstrated that OGD by itself has profound effects on gene expression. Pathway enrichment analysis identified multiple pathways relating to cell survival and inflammation including “neuroactive ligand-receptor interaction”, “calcium signaling”, “ECM-receptor interaction”, “cAMP signaling”, “MAPK signaling” and “p53 signaling”. Pretreatment with TIA dramatically reduced the number of DEGs after OGD.

Previous studies have shown that 5-HT treatment promotes mitochondrial biogenesis and survival of cortical neurons in vitro [21, 36]. In our study, 5-HT pretreatment was applied 24 h before OGD at the concentration of 50 μM, which falls within the physiologically relevant range [13, 78]. Notably, cells were incubated in serum-free medium lacking 5-HT. Our report shows that 5-HT confers robust neuroprotection against OGD-induced cell death. A considerable amount of studies investigating the regulation and effect of 5-HT receptors under ischemic conditions have mainly centered on 5-HT receptor subtype 5-HT1A [50, 63]. In respect to this, we analyzed mRNA levels of 5-HT1A and -1B after OGD using qRT-PCR. No changes were detected in 5-HT1B mRNA levels, however, OGD decreased the expression of 5-HT1A mRNA transcription, which is in line with the reduction in cell survival after OGD. The increase in cortical cell survival with 5-HT pretreatment conclusively demonstrates that 5-HT plays a neuroprotective role against OGD.

We decided to treat cortical neurons with CIT and FLU, which are among the most commonly prescribed SSRIs. In addition to these two SSRIs, we included TIA, an atypical antidepressant that has been reported to act, among other things, as an SSRE [14]. CIT and FLU have been shown to be neuroprotective in OGD-injured cortical neurons cultured with LPS-stimulated microglia [17]. We did not observe any statistically significant effects of either CIT or FLU on cell viability of OGD-injured cortical neurons in the absence of microglia. This result leads us to speculate that both SSRI antidepressants primarily exert their protective effects indirectly by decreasing microglial-mediated neurotoxicity, which is not reflected in our experimental setup. Considering that SSRIs increase the availability of 5-HT, we expected an additive effect when CIT and FLU were co-administered with 5-HT. However, cell survival was only slightly improved, and this effect did not reach statistical significance. Equally surprising, pretreatment with the SSRE TIA resulted in significantly improved survival of cortical neurons after OGD. The neuroprotective effect of TIA appeared to be stronger than the effect of 5-HT alone, and the combination of TIA and 5-HT resulted in even greater protection. Our results are consistent with a previous study [32], which demonstrated that TIA was neuroprotective against the neurotoxicity of staurosporine and doxorubicin in primary neurons, whereas other antidepressants, including FLU and CIT, were not.

Based on our finding that TIA exerted the strongest neuroprotective effect, we further analyzed the influence of OGD and TIA on mRNA transcription and lipid peroxidation. The qRT-PCR studies revealed increased expression of *Trp53*, *Serpine1* and *Hif1a* at 6 h after OGD. Transcription factor p53 was previously shown to accumulate during brain ischemia and to trigger apoptosis [30, 79]. Further, a recent study suggested that Serpine1 acts as a main chemotaxis factor for inflammatory neutrophil migration into the ischemic penumbra, and its overexpression in ischemic stroke appears to worsen neuronal damage [56]. In our study, increased mRNA levels of p53 as well as Serpine1 were reduced in TIA-treated neurons. Notably, the magnitude of reduction in *Serpine1* expression by TIA was particularly striking. Notwithstanding, TIA treatment did not demonstrate efficacy in decreasing the elevated expression of *Hif1a.* Additionally, an increase in the expression of various metalloproteinases in neuroinflammation and stroke has been reported in previous studies [80]. However, we here found no change in the expression of *Mmp3* and *Adamts7* in either the OGD-VEH or OGD-TIA conditions.

Serpine1 as a plasminogen activator inhibitor may be one of the key therapeutic targets during the early reperfusion of occluded vessels. Oxidative injury after reperfusion caused by oxygen-derived free radicals could be as deleterious as the initial damage caused by the occlusion itself. Ferroptosis, a unique form of cell death caused by oxidative stress may constitute a significant component of the pathophysiology of stroke together with other mechanisms such as apoptosis, autophagy, necroptosis and pyroptosis [18, 60, 62, 71]. Hence, for an in vitro model of stroke, we kept the reoxygenation time as 24 h and detected accumulated lipid peroxidation induced by reactive oxygen species and oxidative stress at 24 h after OGD in cortical cells. The neuroprotective effect of TIA was further supported by our finding that it strongly counteracted the inducing effect of OGD on lipid peroxidation.

The DEGs identified in stroke studies have been shown to be primarily associated with the inflammatory, immune and stress responses, glucose metabolism, neurosignaling and apoptosis [15, 57, 75]. Animal studies of stroke are influenced by many interacting systems such as the surrounding vascular system and the immune system. By contrast, in vitro studies offer a more tightly controlled and precise analysis of cell autonomous transcriptional responses to ischemia. Therefore, we aimed to investigate the effects of TIA pretreatment against ischemia on pure murine cortical neurons by analyzing transcriptional profiles of four experimental groups: CONTROL-VEH, OGD-VEH, CONTROL-TIA and OGD-TIA. To study early changes in gene regulation that could ultimately lead to neuronal damage or neuroprotection, we adhered to a 6-h period of reoxygenation for mRNA analysis instead of 24 h used in the cell viability assays. A comparison of the numbers of DEGs at 6 h post-OGD revealed that with TIA pretreatment, OGD was unable to regulate as many genes as without TIA. The decrease in the number of DEGs enables us to infer that TIA represses the regulation of those genes that may contribute to neuronal damage caused by OGD. The expression heatmap results show no statistically significant difference between the CONTROL-VEH and CONTROL-TIA groups, indicating that under normal physiological conditions, TIA does not have a noticeable impact, but the visible contrast between OGD-VEH and OGD-TIA suggests that TIA may have neuroprotective effects by reversing the differential gene expression changes induced by OGD (Fig. 4F).

Pathway enrichment analysis further showed that several pathways associated with the inflammatory response, cell survival and communication were induced following OGD, but these pathways were largely absent in the presence of TIA. The calcium signaling pathway was the most differentially regulated pathway, suggesting that TIA treatment promotes cell survival by restoring the expression of genes involved in calcium signaling to their normal levels. A previous study reported that the calcium signaling pathway remains constantly regulated throughout various stages of reoxygenation, while other pathways are activated and deactivated at different timepoints following 45 min of OGD in hippocampal neurons [65]. Calcium signaling plays a crucial role in responding to oxidative stress during ischemia. The depletion of energy stores caused by the ischemic insult results in malfunction of ion pumps and the release of toxic levels of glutamate, which, in turn, leads to elevated intracellular calcium concentrations [7, 11, 55]. This, in combination with oxidative injury by reoxygenation, results in excess ROS production and lipid peroxidation, ultimately leading to cell death. Differential expression analysis showed that the majority of the DEGs (for the comparison of OGD and CONTROL) involved in calcium signaling were downregulated following OGD (Supplementary Fig. 4A), resulting in the disturbance of the pathway, which may contribute to neuronal damage through both lipid peroxidation and disruption of neuronal signaling.

Interestingly, our cell viability assays showed that the neuroprotective effect of TIA does not depend on 5-HT, but this does not exclude its potential impact on 5-HT signaling, as RNA-seq showed that OGD failed to downregulate 5-HT receptors in the presence of TIA. 5-HT receptors (Htr2c, Htr4, Htr51, Htr7) that were downregulated following OGD (data not shown) are involved in calcium signaling, with changes in the expression of *Htr4* depicted in Fig. 4G. Numerous studies have suggested that the antidepressant effects of TIA are achieved through its regulation of glutamatergic neurotransmission and synaptic plasticity, which may also contribute to its neuroprotective properties [25, 52, 81]. TIA has been shown to decrease glutamate efflux and inhibit stress-induced neurotoxic glutamate transmission by modulating glutamate receptors through a CaMK2-dependent mechanism [52, 61, 81]. Transcriptomic profiling demonstrated that OGD resulted in downregulation of *Camk2a*, a key regulator of calcium signaling, synaptic plasticity and cognitive function. Again, this downregulation of *Camk2a* was attenuated by TIA treatment. The other key components of the calcium signaling pathway, PMCAs such as *Atp2b3* and *Atp2b4*, and NCXs such as *Slc8a2,* together with *Grin1,* showed similar expression trends to Camk2a in response to OGD and TIA. Downregulation of PMCA and NCX mRNAs following OGD can lead to failure of the Ca^2+^ clearance from the cytosol, exacerbation of Ca^2+^ accumulation, and excitotoxicity [2, 3], but this was again alleviated after pre-treatment with TIA.

Glutamate-mediated neurotoxicity also plays an important role in the pathogenesis of various neurodegenerative diseases [1]. In particular, in Alzheimer's disease (AD), amyloid-β peptide has been shown to lead to an increase in extracellular glutamate levels [5]. This mediates a sustained influx of calcium ions and, similar to OGD, triggers a series of pathological events, including mitochondrial and synaptic dysfunction, increased ROS production, and eventually neuronal death [70]. Therefore, TIA as a glutamatergic modulator may also represent a promising drug for the treatment of neurodegenerative diseases. Interestingly, a recent retrospective observational study in AD patients showed beneficial effects on several cognitive domains after 12 months of treatment with TIA, whereas patients treated with other antidepressants did not show significant improvement in cognitive functions [24]. However, further clinical studies are needed to understand the exact effects of TIA on neurodegenerative diseases independent of the antidepressant effect.

Nearly all the DEGs associated with p53 signaling were upregulated following OGD. Inhibition of p53 has been demonstrated to be protective against ischemic damage [43, 67]. TIA reduced the elevated expression of essential components of p53 signaling, thereby hindering the pathway in OGD-TIA vs. CONTROL-TIA (Fig. 4E). Of these components, in addition to *Trp53* and *Serpine1*, *Fas, Tnfrs10b* (belongs to Death Receptor 5 (DR5) family) and *Casp8* as part of the p53/Fas/DR5/CASP8 axis, play a critical role that ultimately triggers apoptosis (Supplementary Fig. 4B). Fas has also been implicated in further inducing the generation of ROS during conditions of oxidative stress [16, 76]. Moreover, it has been reported that the activation of caspase 3 leads to the cleavage and degradation of PMCAs causing a further buildup of calcium ions inside the cell [64]. This highlights a connection between p53 and calcium signaling. Therefore, we can propose that TIA, by reducing the activation p53/Fas/DR5/CASP8 axis, which is upstream of caspase 3 activation, and also by reversing the decrease in PMCA, NCX, CAMK mRNA levels, may protect against both apoptosis and ferroptosis that occur following OGD. However, future studies will be necessary to fully understand the precise effect of TIA on the regulation of glutamate toxicity, inflammation, ferroptosis and apoptosis induced by ischemia, as well as on the associated pathways.

To our knowledge, this is the first report of neuroprotective effects of antidepressant pharmocotherapy against ischemic injury in pure murine cortical neurons. Our study provides insights into processes that lead to neuroprotection through preconditioning with TIA pretreatment, which can serve as a basis for developing new approaches for protecting the brain from ischemia.

### Supplementary Information

Below is the link to the electronic supplementary material.

Supplementary Figure 1. Concentration-dependent toxicity of CIT, FLU, and TIA. Cell viability of primary neuronal cultures treated with increasing concentrations (0.1, 1, 10, 100 μM) of CIT (A), FLU (B), and TIA (C) for 24 h. MTT values were normalized to the CONTROL-VEH condition. Kruskal-Wallis test with Dunn's multiple comparisons test; *p < 0.05 treatment versus VEH, n=4 biological replicates per group. 

Supplementary Figure 2. OGD-dependent expression changes of Htr1a and Htr1b. Primary neuronal cultures were subjected to 2-h OGD. MRNA transcription of 5-HT receptors Htr1a (A) and Htr1b (B) was studied in CONTROL and OGD cells at 6-h of reoxygenation (normalized to housekeeping gene Reep5). N = 5 biological replicates per group. Unpaired t test. t = 3.689, **p < 0.01, OGD versus CONTROL.

Supplementary Figure 3. Heatmap of sample-to-sample distances and GO analysis. (A) Hierarchical clustering of the samples in the comparisons of OGD-VEH versus CONTROL-VEH, OGD-TIA versus CONTROL-TIA, CONTROL-TIA versus CONTROL-VEH and OGD-TIA versus OGD-VEH was shown as a heatmap function derived using DeSeq2 on normalized read counts. Lowest Euclidean distances are darker blue color (closely related samples) and the highest Euclidean distances are pale green color (distantly related samples). (B-C) Gene ontology (GO) enrichment analysis of the genes differentially expressed in the absence and the presence of TIA. The top biological process GO terms with the most significant p-value in OGD-VEH versus CONTROL-VEH (B) and OGD-TIA versus CONTROL-TIA (C) were obtained.

Supplementary Figure 4. KEGG pathway maps. (A) Calcium signaling pathway as represented by KEGG (pathway: mmu04020). (B) P53 signaling pathway as represented by KEGG (pathway: mmu04115). Differentially expressed genes in OGD-VEH versus CONTROL-VEH and OGD-TIA versus CONTROL-TIA are color coded. Pathway maps are adapted from https://www.kegg.jp/kegg/mapper/ [35].
