# Supplementary figures and images for: The atypical antidepressant tianeptine confers neuroprotection against oxygen–glucose deprivation
Source: Eur Arch Psychiatry Clin Neurosci. 2023 Sep 1;274(4):777–91. doi: 10.1007/s00406-023-01685-9 (PMC11127858; doi:10.1007/s00406-023-01685-9)

## Slide 1
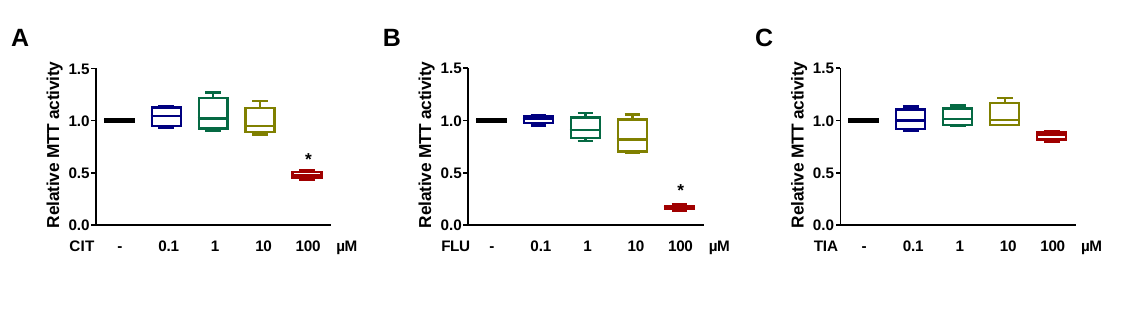

C
B
A

Supplement: Supplementary file 1 — Supplementary Figure 1. Concentration-dependent toxicity of CIT, FLU, and TIA. Cell viability of primary neuronal cultures treated with increasing concentrations (0.1, 1, 10, 100 μM) of CIT (A), FLU (B), and TIA (C) for 24 h. MTT values were normalized to the CONTROL-VEH condition. Kruskal-Wallis test with Dunn's multiple comparisons test; *p < 0.05 treatment versus VEH, n=4 biological replicates per group. [file 406_2023_1685_MOESM1_ESM.pptx]

## Slide 1
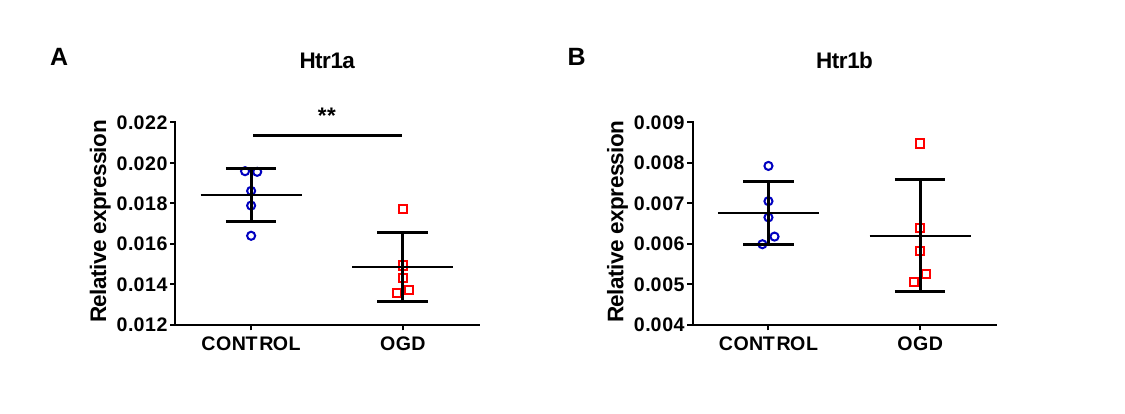

A
B

Supplement: Supplementary file 2 — Supplementary Figure 2. OGD-dependent expression changes of Htr1a and Htr1b. Primary neuronal cultures were subjected to 2-h OGD. MRNA transcription of 5-HT receptors Htr1a (A) and Htr1b (B) was studied in CONTROL and OGD cells at 6-h of reoxygenation (normalized to housekeeping gene Reep5). N = 5 biological replicates per group. Unpaired t test. t = 3.689, **p < 0.01, OGD versus CONTROL. [file 406_2023_1685_MOESM2_ESM.pptx]
