# Supplementary material for: The atypical antidepressant tianeptine confers neuroprotection against oxygen–glucose deprivation
Source: Eur Arch Psychiatry Clin Neurosci. 2023 Sep 1;274(4):777–91. doi: 10.1007/s00406-023-01685-9 (PMC11127858; doi:10.1007/s00406-023-01685-9)
Supplement: Supplementary file 3 — Supplementary Figure 3. Heatmap of sample-to-sample distances and GO analysis. (A) Hierarchical clustering of the samples in the comparisons of OGD-VEH versus CONTROL-VEH, OGD-TIA versus CONTROL-TIA, CONTROL-TIA versus CONTROL-VEH and OGD-TIA versus OGD-VEH was shown as a heatmap function derived using DeSeq2 on normalized read counts. Lowest Euclidean distances are darker blue color (closely related samples) and the highest Euclidean distances are pale green color (distantly related samples). (B-C) Gene ontology (GO) enrichment analysis of the genes differentially expressed in the absence and the presence of TIA. The top biological process GO terms with the most significant p-value in OGD-VEH versus CONTROL-VEH (B) and OGD-TIA versus CONTROL-TIA (C) were obtained. [file 406_2023_1685_MOESM3_ESM.pptx]

## Slide 1
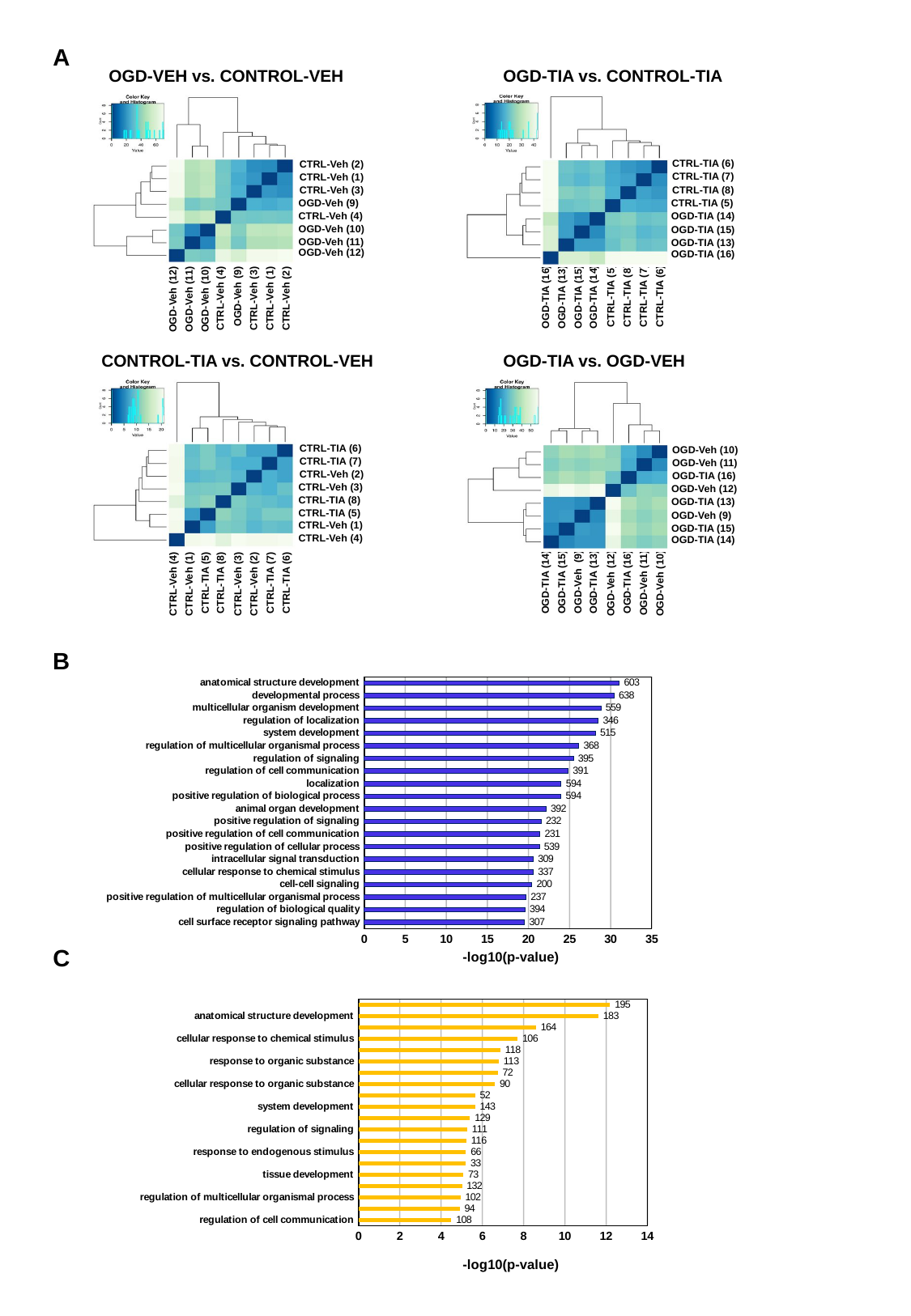

A
OGD-VEH vs. CONTROL-VEH
OGD-TIA vs. CONTROL-TIA
CTRL-TIA (6)
CTRL-TIA (7)
CTRL-TIA (8)
CTRL-TIA (5)
OGD-TIA (14)
OGD-TIA (15)
OGD-TIA (13)
OGD-TIA (16)
CTRL-TIA (7)
OGD-TIA (16)
OGD-TIA (15)
OGD-TIA (14)
CTRL-TIA (5)
CTRL-TIA (8)
CTRL-TIA (6)
OGD-TIA (13)
CTRL-Veh (2)
CTRL-Veh (1)
CTRL-Veh (3)
OGD-Veh (9)
CTRL-Veh (4)
OGD-Veh (10)
OGD-Veh (11)
OGD-Veh (12)
CTRL-Veh (1)
OGD-Veh (12)
OGD-Veh (10)
CTRL-Veh (4)
OGD-Veh (9)
CTRL-Veh (3)
CTRL-Veh (2)
OGD-Veh (11)
OGD-TIA vs. OGD-VEH
CONTROL-TIA vs. CONTROL-VEH
OGD-Veh (10)
OGD-Veh (11)
OGD-TIA (16)
OGD-Veh (12)
OGD-TIA (13)
OGD-Veh (9)
OGD-TIA (15)
OGD-TIA (14)
OGD-Veh (11)
OGD-TIA (14)
OGD-Veh (9)
OGD-TIA (13)
OGD-Veh (12)
OGD-TIA (16)
OGD-Veh (10)
OGD-TIA (15)
CTRL-TIA (6)
CTRL-TIA (7)
CTRL-Veh (2)
CTRL-Veh (3)
CTRL-TIA (8)
CTRL-TIA (5)
CTRL-Veh (1)
CTRL-Veh (4)
CTRL-TIA (7)
CTRL-Veh (4)
CTRL-TIA (5)
CTRL-TIA (8)
CTRL-Veh (3)
CTRL-Veh (2)
CTRL-TIA (6)
CTRL-Veh (1)
B
### Chart
| Category | |
|---|---|
| cell surface receptor signaling pathway | 19.497572880015568 |
| regulation of biological quality | 19.576754126063193 |
| positive regulation of multicellular organismal process | 19.69680394257951 |
| cell-cell signaling | 20.407823242604135 |
| cellular response to chemical stimulus | 20.57839607313017 |
| intracellular signal transduction | 20.585026652029182 |
| positive regulation of cellular process | 21.324221658325914 |
| positive regulation of cell communication | 21.39469495385889 |
| positive regulation of signaling | 21.54821356447571 |
| animal organ development | 22.14146280243036 |
| positive regulation of biological process | 23.958607314841775 |
| localization | 23.97881070093006 |
| regulation of cell communication | 24.835647144215564 |
| regulation of signaling | 25.46344155742847 |
| regulation of multicellular organismal process | 26.12609840213554 |
| system development | 28.129596094720974 |
| regulation of localization | 28.48678239993206 |
| multicellular organism development | 28.815308569182402 |
| developmental process | 30.406713932979542 |
| anatomical structure development | 31.046723663332696 |-log10(p-value)
C
### Chart
| Category | |
|---|---|
| regulation of cell communication | 4.504455662453552 |
| regulation of localization | 4.903089986991944 |
| regulation of multicellular organismal process | 4.943095148663527 |
| cellular developmental process | 5.018181392829336 |
| tissue development | 5.078313524516398 |
| response to wounding | 5.199970640755866 |
| response to endogenous stimulus | 5.2076083105017466 |
| regulation of biological quality | 5.229147988357856 |
| regulation of signaling | 5.266802734893431 |
| cell differentiation | 5.405607449624573 |
| system development | 5.649751981665837 |
| cellular response to oxygen-containing compound | 5.653647025549361 |
| cellular response to organic substance | 6.607303046740334 |
| response to oxygen-containing compound | 6.752026733638194 |
| response to organic substance | 6.795880017344075 |
| animal organ development | 6.876148359032914 |
| cellular response to chemical stimulus | 7.714442690992226 |
| multicellular organism development | 8.607303046740334 |
| anatomical structure development | 11.630784142589857 |
| developmental process | 12.193141970481182 |-log10(p-value)
