# Supplementary material for: The atypical antidepressant tianeptine confers neuroprotection against oxygen–glucose deprivation
Source: Eur Arch Psychiatry Clin Neurosci. 2023 Sep 1;274(4):777–91. doi: 10.1007/s00406-023-01685-9 (PMC11127858; doi:10.1007/s00406-023-01685-9)
Supplement: Supplementary file 4 — Supplementary Figure 4. KEGG pathway maps. (A) Calcium signaling pathway as represented by KEGG (pathway: mmu04020). (B) P53 signaling pathway as represented by KEGG (pathway: mmu04115). Differentially expressed genes in OGD-VEH versus CONTROL-VEH and OGD-TIA versus CONTROL-TIA are color coded. Pathway maps are adapted from https://www.kegg.jp/kegg/mapper/ [35]. [file 406_2023_1685_MOESM4_ESM.pptx]

## Slide 1
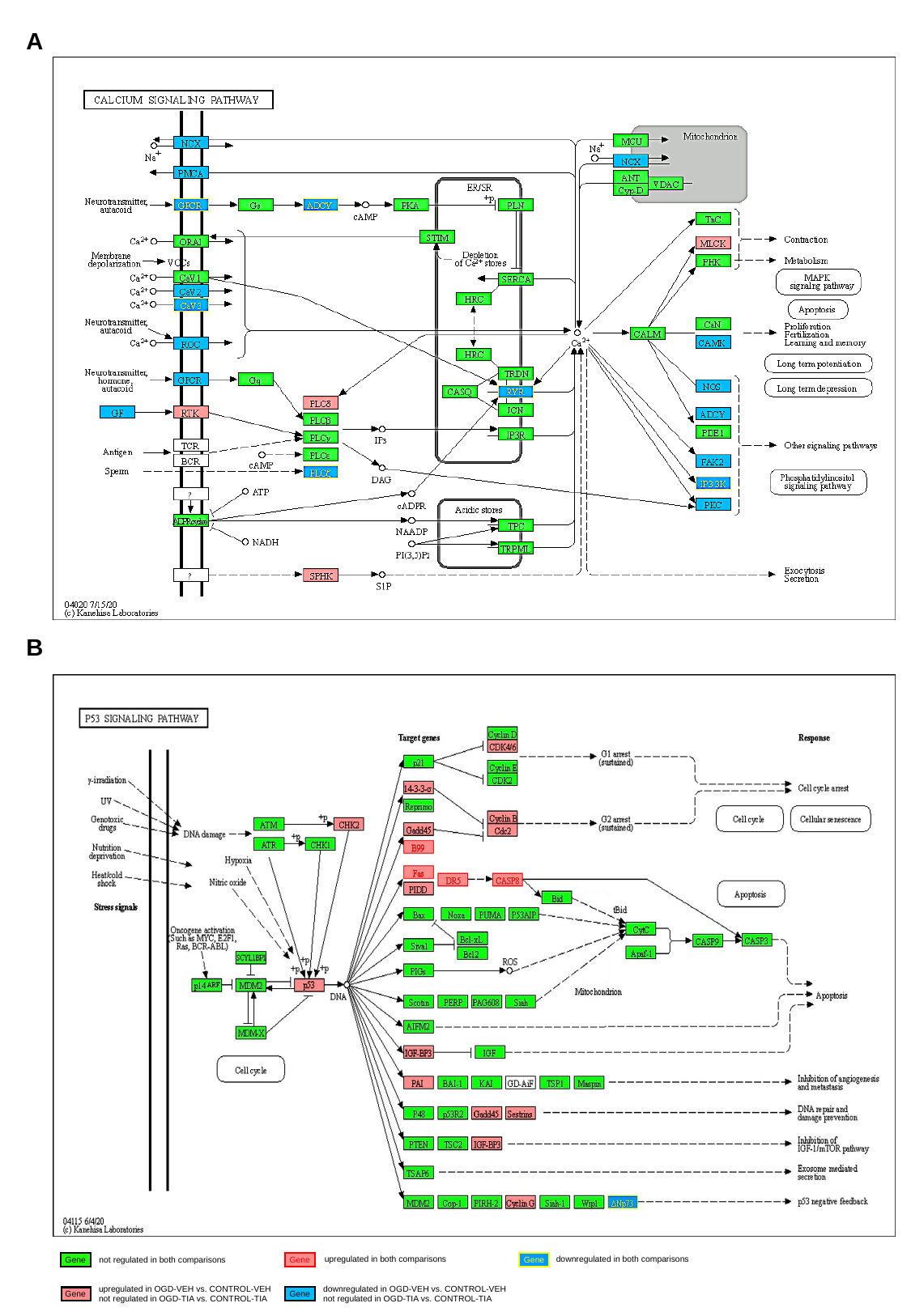

A
B
upregulated in both comparisons
Gene
downregulated in both comparisons
Gene
 not regulated in both comparisons
Gene
upregulated in OGD-VEH vs. CONTROL-VEH
not regulated in OGD-TIA vs. CONTROL-TIA
Gene
downregulated in OGD-VEH vs. CONTROL-VEH
not regulated in OGD-TIA vs. CONTROL-TIA
Gene
